# Histopathological Assessment and Oxidative Biomarker Analysis of Wild Boar Tissues Affected by Ochratoxin A Contamination in the Campania Region, Southern Italy

**DOI:** 10.3390/toxins17090428

**Published:** 2025-08-26

**Authors:** Sara Damiano, Consiglia Longobardi, Evaristo Di Napoli, Valeria Russo, Giuseppe Piegari, Antonio Raffaele, Francesco Ferrucci, Antonio Rubino, Roberto Ciarcia

**Affiliations:** 1 Department of Veterinary Medicine and Animal Production, University of Naples “Federico II”, 80137 Naples, Italy; sara.damiano@unina.it (S.D.); valeria.russo@unina.it (V.R.); giuseppe.piegari@unina.it (G.P.); francescoferrucci92@icloud.com (F.F.); antoruby@virgilio.it (A.R.); rciarcia@unina.it (R.C.); 2 President of the Local Management Hunting Authority (ATC), Province of Avellino, Campania Region, 83100 Avellino, Italy; raffaeleantonio@libero.it

**Keywords:** ochratoxin A, wild boar, kidney, liver, muscle

## Abstract

Ochratoxin A (OTA) is a mycotoxin found in a variety of human foods and in animals. Wild boars are widespread on the European mainland. As they are ubiquitous and feed mainly on a varied diet, they can serve as an excellent bioindicator for OTA research. In recent years, there has been increasing interest in the use of oxidative stress (OS) markers to assess the physiological response of wildlife to environmental stress. Therefore, the aim of this study was to evaluate OS through biochemical assays and morphological changes in liver, kidney and muscle of wild boar that had responded positively to OTA in the Campania region, southern Italy. Endogenous antioxidant enzymes, as well as lipid peroxidation, were quantified using commercially available assay kits. Histological analysis was performed by haematoxylin and eosin staining. Our results indicate that OTA contamination can lead to oxidative stress (OS) and various histopathological changes, primarily affecting the liver and kidneys of OTA-positive wild boars, but not their muscles. Overall, this study highlights the occurrence of OTA contamination in food products, contributing to the broader concern regarding food safety and the potential health risks for humans and animals associated with mycotoxin exposure.

## 1. Introduction

Climate change, in particular the increase in the frequency and intensity of heat waves, has favoured the growth, spread and production of mycotoxins [1]. Mycotoxins are estimated to contaminate approximately 25% of agricultural commodities globally, resulting in annual economic losses that reach several billions of dollars [1,2]. Ochratoxin A (OTA) is among the most toxic compounds within the ochratoxin family, secondary metabolites naturally produced by moulds of the genera *Aspergillus* and *Penicillium* [3]. The mould strains that produce OTA vary depending on the chemical composition of the crop plants and the geographical area. *Penicillium* species, for example, predominantly grow on carbohydrate-rich cereals in temperate climates, whereas *Aspergillus* ones are more commonly associated with a wide variety of foods, particularly those high in fat and protein, mainly in tropical and subtropical regions [4]. OTA is found in a variety of agricultural crops and food, including cereals, pulses, cocoa, milk, meat, spices, liquorice, beer, wine, fruits and nuts [5,6]. It is considered to be an emerging toxic pollutant.

The development of mycotoxicosis is influenced by multiple factors, including genetic, physiological and environmental aspects. In southern Europe the combination of warm temperatures, high humidity, and inadequate grain storage favours the formation of OTA [7]. Although the nephrotoxic effect of OTA is the most studied and important, the toxic effect of this metabolite is not limited to the kidneys, and neurotoxic, immunotoxic, hepatotoxic, teratogenic and carcinogenic effects have also been demonstrated in several animal species [8,9]. Due to the cancer risk for humans, OTA was classified as probably carcinogenic to humans in group 2B (IARC classification) [10]. In humans, OTA has been associated with critical illnesses, such as renal tumours and chronic interstitial nephropathy [11]. As OTA is a molecule with high stability and high protein affinity, especially for albumin, it accumulates in various tissues and organs and can lead to carry-over of contamination. Therefore, it can pass unchanged through the food chain, and the contamination can spread to foods of animal origin such as meat, milk and fermented foods [12]. OTA exhibits species-specific toxicity, with pigs recognised as the most sensitive among mammals [11]. In poultry, ducklings show the highest susceptibility, followed by chickens, turkeys, and quails [13]. The toxicological effects of OTA include marked nephrotoxicity, impairment of growth performance, reduction in egg production, and deterioration of eggshell quality [14].

Regulations on mycotoxins have been enacted in many countries to protect consumers from the harmful effects of these compounds. Various factors play a role in the decision-making process to establish safe limits for mycotoxins. These include scientific factors, e.g., the availability of toxicological and epidemiological data, detailed knowledge of sampling and analysis options and socio-economic aspects [15]. Commission regulation (UE) 2022/1370 contains OTA limits for breakfast cereals, non-alcoholic malt beverages, wheat gluten, date juice, dried herbs, various roots for herbal teas, various seeds, pistachios and cocoa powder. The new limits range from 0.5 µg/kg for processed cereal-based foods for infants and young children to 80 µg/kg for liquorice extract used in drinks and confectionery. Most limits are between 2 and 8 µg/kg for cereal-based products, dried fruit, wine, juices, seeds, pistachios, cocoa and coffee [16]. In Italy, the Ministry of Health has recommended a maximum level of 1 µg/kg OTA in pork and pork-derived products [17].

Climate change, emerging infectious diseases, and anthropogenic disturbances are recognised by the World Health Organization (WHO) as major global public health challenges [18]. In recent years, significant transformations in the natural environment and related human activities have been observed in Italy, particularly in the Campania region. Gaining insight into the physiological responses of wildlife to these environmental stressors is critical for the effective management, monitoring, and conservation of wild animal populations [19].

Among all wild animal species, wild boar appears to have adapted most successfully to environmental and anthropogenic changes (for example, habitat fragmentation, agricultural expansion, and reduced predation pressure) and has consequently become one of the most densely populated free-ranging mammal species in Europe. Therefore, with its demographic increase, the wild boar has become a problem not only for agriculture, but also for health and safety [16]. The wild boar’s widespread distribution and opportunistic omnivorous diet, including starchy plant materials, fungi, and animal-derived food sources, make it a suitable bioindicator species for studies on OTA contamination [20]. In the Campania region, wild boar meat is traditionally used to produce niche products, especially coppa, and salami. Furthermore, OTA can also co-contaminate spices and flavourings added during the processing of cured meats, thus representing an additional source of contamination [21].

Several studies suggest that OTA toxicity can be associated with the induction of oxidative stress (OS), which subsequently leads to DNA damage and the occurrence of mutations [22,23,24,25]. Therefore, the aim of this study was to assess OS in wild boars from the Campania region testing positive for OTA contamination, by measuring specific biochemical markers such as malondialdehyde (MDA), superoxide dismutase (SOD), catalase (CAT), and glutathione peroxidase (GPx). In addition, histological analyses of liver, kidney, and muscle tissues were conducted to identify potential morphological alterations associated with OTA exposure.

## 2. Results

### 2.1. The Effect of OTA on Lipid Peroxidation

**Figure 1:**
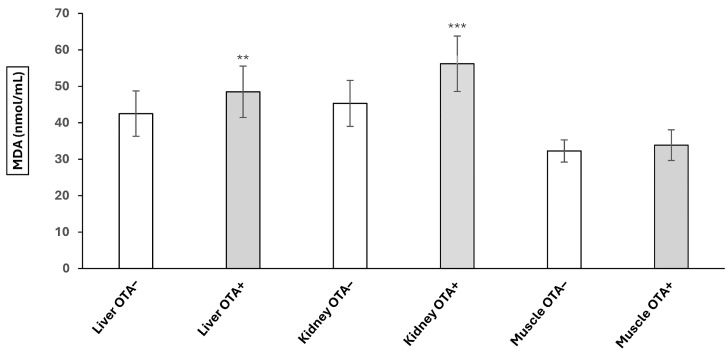
Malondialdehyde (MDA) levels in liver, kidney, and muscle tissues of wild boars (*Sus scrofa*). Comparison between OTA-contaminated samples (OTA+) and OTA-uncontaminated samples (OTA−) (** *p* < 0.01; *** *p* < 0.001).

Malondialdehyde (MDA) levels in liver and kidney tissue were significantly increased in the OTA-contaminated samples (OTA+) compared to the OTA-uncontaminated samples (OTA−). The values increased from 42.49 ± 6.22 (OTA−) to 48.4 8 ± 7.06 (OTA+) in the liver (*p* < 0.01) and from 45.30 ± 6.30 (OTA−) to 56.18 ± 7.61 (OTA+) in the kidney (*p* < 0.01). No statistical changes in MDA levels were observed in the muscle. The MDA value in muscle tissue was 33.84 ± 4.20 in the OTA+ group compared to 32.25 ± 3.04 in the OTA− group (Figure 1).

### 2.2. GPx, CAT and SOD Activities Changes upon OTA Exposure

**Figure 2:**
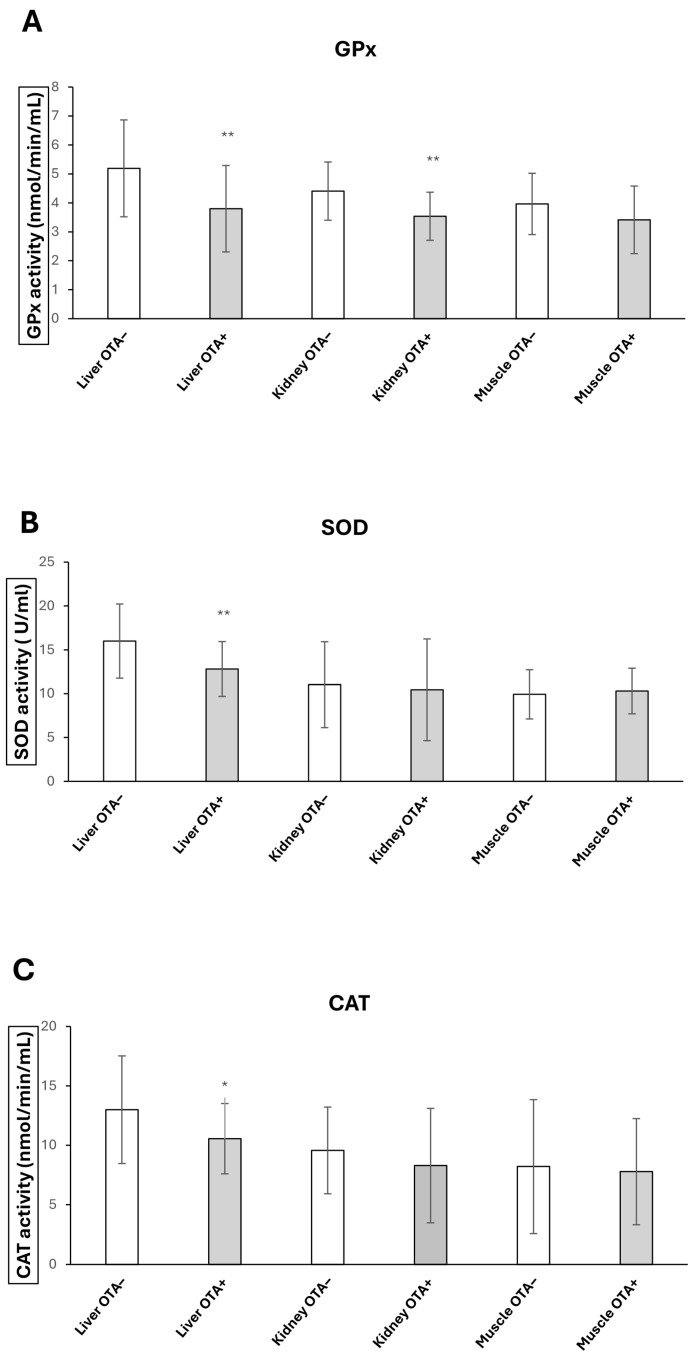
Effects of Ocratoxin A (OTA) on the activities of glutathione peroxidase (GPx) (**A**), superoxide dismutase (SOD) (**B**) and catalase (CAT) (**C**) in the livers (*n* = 26 OTA−; *n* = 26 OTA+), kidneys (*n* = 26 OTA−; *n* = 26 OTA+) and muscles (*n* = 10 OTA−; *n* = 10 OTA+) of wild boars. Comparison between OTA-contaminated group (OTA+) and OTA-uncontaminated group (OTA−). Results are expressed as mean standard deviation (SD). (* *p* < 0.05; ** *p* < 0.01). Data are expressed as nmol/min/mL for GPx and CAT, and as U/mL for SOD.

Glutathione peroxidase (GPx), catalase (CAT) and superoxide dismutase (SOD) activities detected in liver, kidney and muscle tissue of wild boars are shown in Figure 2A–C. GPx activities were significantly decreased in the liver and kidneys of wild boars from the OTA+ group compared to the OTA− group. GPx values in the liver decreased from 5.19 ± 1.67 (OTA−) to 3.79 ± 1.49 (OTA+) (*p* < 0.01) and in the kidney from 4.40 ± 1.00 (OTA−) to 3.53± 0.83 (OTA+) (*p* < 0.01). No significant fluctuations were observed in GPx activity in muscle, with a shift from 3.96 ± 1.05 (OTA−) to 3.41 ± 1.16 (OTA+). SOD activities were significantly decreased in the liver of wild boars from 15.99 ± 4.22 (OTA−) to 12.80 ± 3.13 (OTA+) (*p* < 0.01). SOD activity in the kidney and muscle tissues of OTA+ wild boars did not differ significantly from that of the OTA− group, with values shifting from 11.02 ± 4.90 to 10.43 ± 5.80 in the kidney, and from 9.92 ± 2.80 to 10.30 ± 2.60 in the muscle, respectively. Conversely, CAT activity showed a significant reduction in the liver of OTA+ animals compared to OTA− (12.99 ± 4.53 vs. 10.55 ± 2.96; *p* < 0.05). No significant changes were observed in the kidney and muscle, where CAT values shifted from 9.56 ± 3.65 to 8.29 ± 4.81, and from 8.21 ± 5.63 to 7.78 ± 4.46, respectively.

### 2.3. Histopathological Findings of Liver, Kidneys and Muscles

**Figure 3:**
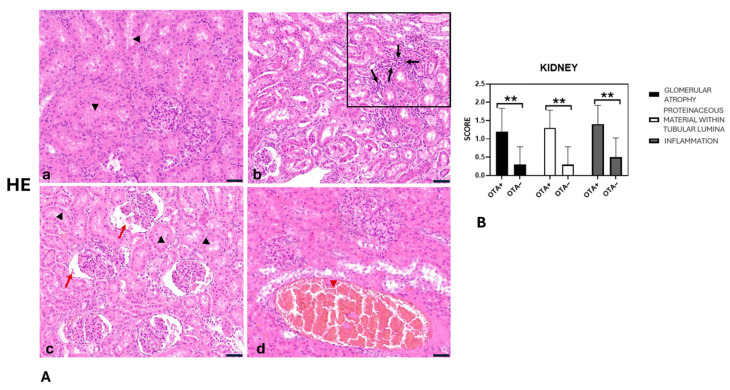
(**A**) Wild boar, kidney, hematoxylin and eosin stain (HE). (**a**) Kidney of OTA− group appeared normal, with a mild accumulation of intratubular proteinaceous material (black arrowhead). (**b**–**d**) Kidneya of the OTA+ group showed a mild-to-moderate, multifocal lymphoplasmacytic interstitial inflammatory infiltrate (**b**) (black arrow). The tubules’ lumen contained mild-to-moderate proteinaceous material (black arrowhead), and mild-to-moderate glomerular atrophy, with an increase in the Bowman’s space (**c**) (red arrow). In addition, we observed vascular congestion (**d**) (red arrowhead). (**B**) Severity scores for glomerular atrophy, presence of proteinaceous material in the tubular lumen, and inflammation. Asterisks represent statistically significant differences between groups (** *p* < 0.01 OTA+ vs. OTA−). Original magnification, 200×, and higher magnification, 400×. Scale bars = 20 µm.

In the OTA− group, kidney morphology appeared largely normal, with only a slight accumulation of intratubular proteinaceous material observed in 3 out of 10 samples. Mild, scattered lymphocytic infiltration within the interstitial space was present in 5 out of 10 cases, while mild glomerular atrophy and hyperaemia were detected in 3 and 4 cases, respectively (Figure 3A,B).

On the other hand, the kidneys of the OTA+ group showed a mild (6 out of 10) to moderate (4 out of 10) multifocal lymphoplasmacytic interstitial inflammatory infiltrate. The lumen of the tubules contained mild (7 of 10) to moderate (3 of 10) proteinaceous material. Glomerular atrophy with enlargement of Bowman’s space was classified as mild (6 out of 10) to moderate (3 out of 10). The tubular epithelial cells were often atrophic or degenerated. We observed hyperaemia in all cases (Figure 3A,B). Hystopathological score values are reported in Table 1.

**Figure 4:**
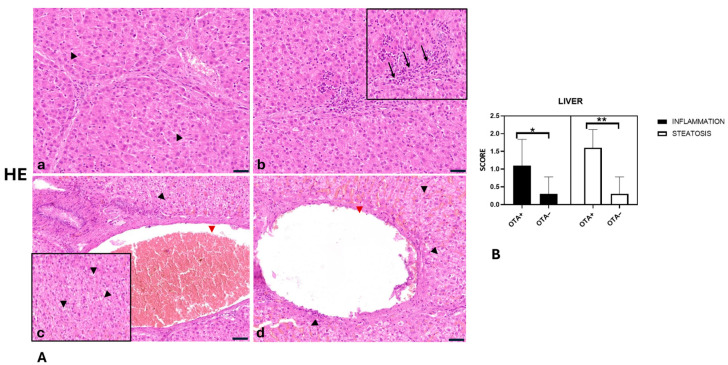
(**A**) Wild boar, liver, hematoxylin and eosin stain (HE). (**a**) Livers of the OTA− group showed a few disseminated hepatocytes with swollen cytoplasm (black arrowhead). (**b**) Livers of the OTA+ group showed mild-to-moderate multifocal, lymphoplasmacytic inflammation (**b**) (black arrow). (**c**,**d**) Livers of the OTA+ group showed mild-to-moderate degenerated hepatocytes with optically empty cytoplasm containing small, rounded vacuoles that sometimes move the nucleus to the periphery (microvacuolar steatosis; black arrowhead); dilation of the portal (**c**) (red arrowhead) and the centrolobular vein (**d**) (red arrowhead). (**B**) Severity scores for inflammation and steatosis. Asterisks represent statistically significant differences between groups (** *p* < 0.01 OTA+ vs. OTA− and * *p* < 0.05 OTA+ vs. OTA−). Original magnification, 200×, and higher magnification, 400×. Scale bar = 20 µm.

The livers of the OTA− group did not show significant pathologic findings; only in three out of ten did we observe a mild infiltrating inflammatory cell, and a few disseminated hepatocytes showed swollen cytoplasm. Rarely did we observe dilatation of the portal and centrilobular veins (Figure 4A,B).

Differently, livers from the OTA+ group showed mild (in 5 out of 10) to moderate (in 3 out of 10), multifocal, lymphoplasmacytic inflammation. Morphological evaluation of livers revealed mild (4 out of 10) to moderate (6 out of 10) degenerated hepatocytes with optically empty cytoplasm containing small, rounded vacuoles that sometimes cause nuclear displacement (microvacuolar steatosis). We observed a dilation of the portal and centrilobular veins in most of the cases examined (mild in 5 out of 10 and moderate in 4 out of 10). Statistical analysis showed statistically significant differences in liver inflammation between the OTA− group and the OTA+ group (*p* < 0.05) [*p* = 0.0270]. Also, statistically significant differences in steatosis were found between the OTA− group and the OTA+ group (*p* < 0.05) [*p* = 0.0004] (Figure 4A,B). Histopathological score values are reported in Table 2.

**Figure 5:**
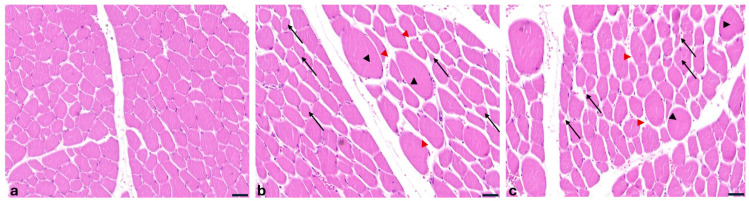
Wild boar, muscle, hematoxylin and eosin stain (HE). (**a**) The muscles of OTA− group did not show significant pathological changes. (**b**,**c**) Muscle tissues from the OTA+ group showed variability in the myofiber diameter, fibres reduced in size (**b**,**c**) (atrophy; black arrow) with an angular profile, increased endomysial spaces (**b**,**c**) (red arrowhead), and hypertrophic myofibers (**c**) (black arrowhead). Original magnification, 200×. Scale bar = 20 µm.

Muscles from OTA− wild boars showed no significant pathological alterations. However, morphological assessment of OTA+ muscle samples revealed mild variability in myofiber diameter, size reduction (atrophy) with angular profiles, widening of endomysial spaces, and occasional myofibers exhibiting compensatory hypertrophy (degeneration) (Figure 5).

To summarise:

- Glomerular atrophy was significantly greater in the OTA+ group compared with the OTA− group (*p* = 0.0080).
- Proteinaceous material in tubular lumina was significantly more abundant in the OTA+ group than in the OTA− group (*p* = 0.0013).
- Kidney inflammation was significantly higher in the OTA+ group compared with the OTA− group (*p* = 0.0050).
- Liver inflammation was significantly greater in the OTA+ group compared with the OTA− group (*p* = 0.0270).
- Liver steatosis was significantly more pronounced in the OTA+ group compared with the OTA− group (*p* = 0.0004).

## 3. Discussion

In recent years, there has been increasing interest in the use of oxidative stress (OS) markers to assess the physiological response of wildlife to environmental stresses, as they are stable and easy to quantify [26,27]. OS is an imbalance between ROS and the cellular mechanisms responsible for antioxidant defence [28]. The relationship between OTA and its toxicity in wild boars has not been extensively studied, with most of the literature focussing on its nephrotoxic effects and carcinogenic potential [29,30]. Given the increasing wild boar population and their growing role in meat production, there is a need to investigate OTA toxic effects on edible tissues such as muscle, liver and kidney. This study focussed on wild boars from the province of Avellino, which has the highest density of this species in the Campania region. Mycotoxins are known to generate OS by promoting the formation of free radicals. OTA is recognised for inducing OS mainly via disrupting mitochondrial function, specifically the electron transport chain, resulting in electron leakage and the consequent generation of superoxide anions and other ROS. Moreover, OTA may interfere with antioxidant enzymes, hence exacerbating ROS accumulation [31]. In this study, MDA levels and the endogenous antioxidant system, consisting of enzymatic antioxidants such as SOD, CAT, and GPx were analysed, together with the histopathological findings of liver, kidney and muscle tissue from wild boars (*Sus scrofa*), identified as OTA-positive in our previous work [32]. Antioxidant enzymes play a crucial role in the neutralisation of ROS and the maintenance of cellular homeostasis [33]. Our results showed a significant increase in MDA levels in liver and kidney tissuea of OTA-positive wild boars compared to non-contaminated animals, indicating increased lipid peroxidation by increased hydroxyl radicals (OH) and superoxide anions (O_2_^−^), which can alter the molecular properties of lipids [34], consistent with previous studies in rodents [35] and piglets [36]. The lack of significant changes in MDA levels in muscle suggests that liver and kidney are the primary targets for OTA-induced oxidative damage in wild boars, probably due to their crucial role in metabolism and detoxification. Our analysis of enzymatic antioxidant defences revealed a significant decrease in the activities of GPx, SOD, and CAT in the liver of OTA+ wild boars, with GPx activity significantly reduced also in the kidneys. These enzymes are key components of the body’s antioxidant system that work synergistically to neutralise ROS and maintain cellular homeostasis [36]. The observed reduction in their activities indicates an impairment of antioxidant capacity in response to OTA exposure, making cells more susceptible to oxidative damage. Although no direct studies are available on wild boars, parallelism with pigs and other experimental models suggests that chronic exposure to OTA in their environment may determine a persistent decline in hepatic antioxidant defences [37]. In fact, the impairment of the antioxidant system could be the cause of the lipid peroxidation and the observed steatosis. This establishes a perpetuating cycle of damage and disease progression, which can further aggravate hepatic injury and could potentially contribute to comorbid conditions [38]. In addition, the increase in MDA and decrease in GPx activity could be due to OTA alteration in ferroptosis activity, a form of cell death associated with the accumulation of iron and lipid peroxides [39,40,41]. Moreover, OTA has been shown to suppress the activity of the transcription factor Nrf2 (nuclear factor erythroid 2–related factor 2), a key regulator of cellular antioxidant defences, in both in vitro [42] and in vivo [43] models. Since Nrf2 nuclear translocation controls the expression of genes encoding antioxidant enzymes, including SOD, CAT, and GPx, its downregulation by OTA can lead to reduced synthesis and activity of these enzymes. This mechanism, combined with OTA-induced depletion of essential cofactors [8], may further compromise the antioxidant defence capacity of liver and kidney tissues. The organ-specific differences observed on antioxidant enzymes, with the liver showing a greater reduction in antioxidative defence than the kidney, emphasises the central role of the liver in xenobiotic metabolism and detoxification, and its susceptibility to mycotoxin-induced oxidative damage. In domestic pigs, considered the physiological analogues of wild boars, multiple isoforms of the CYP450 system participate in the biotransformation of OTA, i.e., CYP1A2, CYP2C, CYP3A [44,45]. These enzymes catalyse the biotransformation of OTA in less toxic compounds than the parent one. It is reasonable to propose that in wild boars, active hepatic metabolism, linked to the functional expression of CYP isoforms, plays a role in decreasing the bioavailability of OTA to peripheral tissues, such as skeletal muscle. This mechanism, together with the strong binding of OTA to albumin [46], may explain the absence of detectable damage in muscle tissue. However, subtle effects on protein turnover and mitochondrial function cannot be excluded, possibly contributing to the observed mild atrophic changes without progressing to irreversible injury [47].

Histological analysis of the kidneys of wild boars classified in the OTA+ exhibited substantial deposits of proteinaceous material, accompanied by inflammation and atrophy, clear indicators of nephrotoxicity induced by OTA [30]. This is in accordance with OTA’s direct effect on the proximal tubules and glomeruli, causing tubular degeneration, glomerulosclerosis [48].

Although OTA-contaminated muscle tissues do not seem to exhibit direct toxic effects, their presence raises significant concerns regarding the potential entry of OTA into the food chain. Wild boars, due to their omnivorous feeding habits and foraging behaviour, can bioaccumulate environmental contaminants such as mycotoxins, acting as sentinels of ecosystem contamination. Consequently, consumption of meat and offal from these animals may represent a possible cause of exposure for humans, especially in rural and hunting communities where game meat is regularly consumed and sometimes shared within local markets. This scenario highlights the complexity of food chain safety, as the presence of OTA in wild animal products could also affect domestic animals when offal or by-products are used as feed ingredients. These findings underscore the importance of implementing preventive strategies to minimise OTA contamination along the entire food chain, from environmental monitoring to food safety control at the point of consumption. Given the documented nephrotoxic, hepatotoxic, and carcinogenic potential of OTA, the European Union has established maximum limits for its concentration in food and feed (EU Regulation 1370/2022) to protect consumer health. Strengthening monitoring programmes and risk assessments for OTA in wildlife products may therefore be warranted. Therefore, this study underlines the importance of assessing the risks of OTA contamination beyond animal husbandry, especially in game meat.

Our samples originate from wild boars hunted in their natural habitat, and this condition offers a unique ecological perspective on OTA exposure in free-ranging populations, while imposing significant analytical limitations. In particular, the highly labile and sensitive quantification of ROS [49] make it challenging to obtain other reliable and representative measurements in field-collected samples. To address these limitations, future studies could employ experimental models, where post-mortem intervals and sample handling can be regulated. Alternatively, implementing field biobanking strategies and training hunters in proper sample preservation techniques could be a viable option.

## 4. Conclusions

This study provides novel data on the impact of OTA in wild boars from the Campania region (Southern Italy) and its potential impact on human health. OTA was associated with significant histopathological changes in the liver and kidney, as well as alterations in OS markers. Although no relevant lesions were detected in skeletal muscle, these findings support the hypothesis that wild boar meat can act as a potential source of OTA exposure for humans, particularly in areas where game meat is commonly consumed. This concern is especially relevant in context where muscles is not the sole edible portion consumed and where wild boar meat is regarded as a valuable and highly prized source of nutrition.

Within a One Health framework integrating human, animal, and environmental health, monitoring the epidemiological patterns of OTA occurrence in wild animals is essential. Establishing targeted environmental surveillance plans can enhance public awareness and inform food safety policies, particularly for game meat, where no maximum residue limits (MRLs) or acceptable daily intake (ADI) values for OTA have yet been defined. This study supports the hypothesis that animal-derived products, including wild boar meat, may serve as a potential source of OTA exposure for humans, and that environmental variability may affect OTA levels in wild boar diets. The consumption of wild meat, when properly monitored, offers an ethical and sustainable option that supports biodiversity management and the valorisation of traditional local products, while contributing to reduced CO_2_ emissions and lower land and water use. Wild game meat also originates from animals that are neither vaccinated nor pharmacologically treated, offering an additional advantage in terms of natural production systems.

## 5. Materials and Methods

### 5.1. Ethics Statement

For this study, approval from an Ethics Committee was not required because the animals were legally hunted in their natural habitat by licenced hunters, following the 2021–2022 annual hunting plan approved by the province of Avellino, Campania region, Italy.

### 5.2. Study Area: Wild Boar Population Density in Avellino, Italy

This study analyses free-ranging wild boars (*Sus scrofa*) in the province of Avellino, located in the Campania Region of Southern Italy. Avellino is characterised by diverse topography, encompassing both mountainous and lowland areas, which influence the distribution and density of wild boar populations. The region lies approximately at 41°′ N, 14°′ E, with altitude variations ranging from 300 m above sea level (m.a.s.l.) in the lower valleys to over 1400 m.a.s.l. in mountainous zones, such as the peaks of Monte Terminio and Monte Partenio [50].

Covering an area of approximately 28,000 hectares, Avellino’s landscape consists of limestone mountain formations, dense forests, and agricultural zones, offering a favourable environment for wild boars. The Mediterranean climate, characterised by warm summers and humid winters, contributes to abundant food resources, including acorns, tubers, fungi, and cultivated crops, further supporting the expansion of the wild boar population.

**Figure 6:**
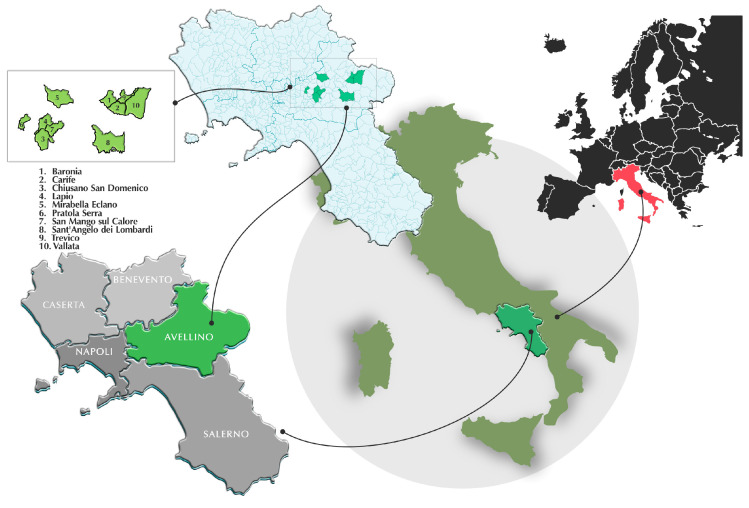
Cartographic representation of the province of Avellino (Campania region, Southern Italy) delineating hunting zones (in green) designated for wild boar (*Sus scrofa*) sampling. The map was created with Affinity Designer 2, version 2.6.3.3322-Serif (Europe) Ltd. (Nottingham, UK).

Given the increasing presence of wild boars in the province, understanding their density and movement patterns is crucial for wildlife management and ecological balance. The study examines how altitude and habitat variation influence population density and evaluates environmental factors affecting the distribution of wild boars within this region. Figure 6 illustrates the hunting areas involved in this study.

### 5.3. Sampling of Free-Ranging Wild Boar

The sampling was conducted following the methodology described in our previous study [32], which reported the presence of OTA in 35.1% (26 out of 74) of the wild boars analysed using HPLC-FLD. Specifically, OTA was detected in 26 livers, 25 kidneys, and 10 muscle samples (biceps femoris). The concentrations of OTA ranged from 0.25 to 2.67 μg/kg in kidneys, from 0.03 to 2.13 μg/kg in livers, and from 0.03 to 3.8 μg/kg in muscles. For this research, convenience sampling approach was utilised. Aliquots of each organ collected from wild boars were either frozen at −80 °C for enzymatic activity assays or fixed in 10% neutral-buffered formalin for histological analysis.

### 5.4. Analysis of Malondialdehyde (MDA) and Antioxidant Enzyme Activities

One gram of each liver, kidney and muscle sample was rinsed with phosphate-buffered saline (PBS) to remove blood cells and clots, then divided into different aliquots. Samples were homogenised on ice using a tissue homogeniser (Tissue Lyser, Qiagen, Milano, Italy) and centrifuged at speeds appropriate for each specific assay. The resulting supernatants were stored at −80 °C.

Lipid peroxidation was assessed by quantifying MDA following the method described by Gassó et al. [49]. The optical density (OD) of the MDA–TBA (thiobarbituric acid) adducts was measured at 535 nm using a spectrophotometer (Glomax Multi detection system, Promega, Milano, Italy). The data were expressed as nmol MDA/mL.

Glutathione Peroxidase Assay Kit, Superoxide Dismutase Assay Kit and Catalase Assay Kit were purchased from Cayman Chemical (Ann Arbor, MI, USA). All other chemical reagents were purchased from Sigma (Milan, Italy).

Glutathione peroxidase (GPx) is the general name of an enzyme family with peroxide activity; its biochemical function is the reduction of lipid hydroperoxides to the corresponding alcohols and the reduction of free hydrogen peroxide to water. The resulting samples were then tested according to the manufacturer’s instructions, the absorbance was read at 340 nm using a spectrophotometer (Glomax Multi detection system, Promega, Milano, Italy) and the results were expressed as nmol/min/mL.

Superoxide dismutase (SOD) is an important antioxidant in almost all cells exposed to ROS generated by cellular immune responses. The resulting samples were then tested according to the manufacturer’s instructions, and the absorbance was measured at 460 nm using a spectrophotometer, and the results were expressed as U/mL.

Catalase (CAT) catalyses the decomposition of hydrogen peroxide produced in the damaged tissue into water and oxygen. The resulting samples were then tested according to the manufacturer’s instructions, and the absorbance was read at 540 nm spectrophotometrically and the results were expressed in nmol/min/mL.

### 5.5. Histopathological Examinations

Samples of boars’ liver, kidney and skeletal muscle (biceps femoris) were fixed for 24 h in 10% neutral buffered formalin and embedded in paraffin. Serial sections of 3–5 μm thickness were stained with haematoxylin and eosin and then evaluated, scanned and photographed using the Pannoramic II scanner (3DHISTECH, The Digital Pathology Company, Övutca 3, 1141 Budapest, Hungary).

The OTA− and OTA+ groups included 10 liver, 10 kidney and 10 skeletal muscle (biceps femoris) tissues, selected as representative of each group based on the presence and severity of macroscopic alterations. A histological scoring system was used that considered the most representative lesions in liver, kidney and muscle tissue.

In the kidneys, haematoxylin and eosin-stained sections were used to assess glomerular atrophy, inflammation and the presence of proteinaceous material within the tubular lumen. Renal lesions were assessed by examining at least 10 microscopic fields at 20× magnification using established histological scoring systems. In particular, inflammation was graded as follows: Score 0 (absent), no foci of inflammation; Score 1 (mild), <2 foci per 20× field; score 2 (moderate), 2–4 foci per 20× field; and Score 3 (severe), >4 foci per 20× field [51,52]. The presence of proteinaceous material in the tubule lumen and glomerular atrophy were scored from 0 to 3 (0 = absent; 1 = mild; 2 = moderate; 3 = severe) [53]. In addition, the presence or absence of hyperaemia was recorded for each case.

Sections stained with haematoxylin and eosin were used in the livers to assess inflammation and steatosis. Liver lesions were assessed by examining at least 10 microscopic fields at 20× magnification using an established histological scoring system. In particular, inflammation was graded as follows: Score 0 (absent), no foci of inflammation; Score 1 (mild), <2 foci per 20× field; Score 2 (moderate), 2–4 foci per 20× field; and Score 3 (severe), >4 foci per 20× field system [54,55]. The extent of steatosis was categorised as follows: Score 0, <5% of hepatocytes; Score 1 (mild), 5–33%; Score 2 (moderate), >33–66%, Score 3 (severe), >66% [51,52]. In addition, the presence or absence of a portal vein and centrilobular vein dilatation was recorded for each case.

In skeletal muscle, sections stained with haematoxylin and eosin were used to assess the morphological aspect of the muscle myofibers.

### 5.6. Statistical Analysis

Statistical analyses of enzymatic activities and malondialdehyde were performed using GraphPad (version 8.0; GraphPad Software Inc., San Diego, CA, USA). The Shapiro–Wilk test and the Kolmogorov–Smirnov tests were used to determine the normality of the data distribution. For histological evaluations, differences between the OTA− and OTA+ groups were determined with an unpaired t-test, while differences between the means of the individual histological semi-quantitative scores were determined with the Mann–Whitney U test. Values of *p* < 0.05 were considered significant.

## Figures and Tables

**Table 1:** Severity scores of glomerular atrophy, inflammation and proteinaceous material in the kidneys of OTA+ and OTA− wild boars.

KIDNEY	Score 0		Score 1		Score 2		Score 3	
	OTA+	OTA−	OTA+	OTA−	OTA+	OTA−	OTA+	OTA−
Glomerular atrophy	1	7	6	3	3	0	0	0
Inflammation	0	5	6	5	4	0	0	0
Proteinaceous material within tubular lumina	0	7	7	3	3	0	0	0

**Table 2:** Severity scores for inflammation and steatosis in the livers of OTA+ and OTA− wild boars.

LIVER	Score 0		Score 1		Score 2		Score 3	
	OTA+	OTA−	OTA+	OTA−	OTA+	OTA−	OTA+	OTA−
Inflammation	2	7	5	3	3	0	0	0
Steatosis	0	7	4	3	6	0	0	0

## Data Availability

The original contributions presented in this study are included in the article. Further inquiries can be directed to the corresponding authors.

## Abbreviations

The following abbreviations are used in this manuscript:

OTA	Ochratoxin A
MDA	Malondialdehyde
GPx	Glutathione peroxidase
SOD	Superoxide dismutase
CAT	Catalase
WHO	World Health Organisation
MRLs	Maximum residue limits

## References

[1] Mesfin A., Lachat C., Vidal A., Croubels S., Haesaert G., Ndemera M., Okoth S., Belachew T., Boevre M., De Saeger S. (2022). Essential descriptors for mycotoxin contamination data in food and feed. Food Res. Int..

[2] Hao W., Li A., Wang J., An G., Guan S. (2022). Mycotoxin Contamination of Feeds and Raw Materials in China in Year 2021. Front. Vet. Sci..

[3] Malir F., Ostry V., Pfohl-Leszkowicz A., Malir J., Toman J. (2016). Ochratoxin A: 50 Years of Research. Toxins.

[4] Heussner A.H., Bingle L.E. (2015). Comparative Ochratoxin Toxicity: A Review of the Available Data. Toxins.

[5] Wu F., Groopman J.D., Pestka J.J. (2014). Public health impacts of foodborne mycotoxins. Annu. Rev. Food Sci. Technol..

[6] Bui-Klimke T.R., Wu F. (2015). Ochratoxin A and human health risk: A review of the evidence. Crit. Rev. Food Sci. Nutr..

[7] van Egmond H.P., Schothorst R.C., Jonker M.A. (2007). Regulations relating to mycotoxins in food: Perspectives in a global and European context. Anal. Bioanal. Chem..

[8] Kőszegi T., Poór M. (2016). Ochratoxin A: Molecular Interactions, Mechanisms of Toxicity and Prevention at the Molecular Level. Toxins.

[9] Rhouati A., Yang C., Hayat A., Marty J.L. (2013). Aptamers: A promising tool for ochratoxin A detection in food analysis. Toxins.

[10] IARC (1993). Ochratoxin A. IARC Monogr. Eval. Carcinog. Risks Hum..

[11] Liu W.-C., Pushparaj K., Meyyazhagan A., Arumugam V.A., Pappuswamy M., Bhotla H.K., Baskaran R., Issara U., Balasubramanian B., Khaneghah A.M. (2022). Ochratoxin A as an alarming health threat for livestock and human: A review on molecular interactions, mechanism of toxicity, detection, detoxification, and dietary prophylaxis. Toxicon.

[12] Reddy L., Bhoola K. (2010). Ochratoxins-food contaminants: Impact on human health. Toxins.

[13] Prior M.G., Sisodia C.S., O’Neil J.B. (1976). Acute oral ochratoxicosis in day-old White Leghorns, turkeys and Japanese Quail. Poult. Sci..

[14] Kuiper-Goodman T., Hilts C., Billiard S.M., Kiparissis Y., Richard I.D., Hayward S. (2010). Health risk assessment of ochratoxin A for all age-sex strata in a market economy. Food Addit. Contam. Part A Chem. Anal. Control Expo. Risk Assess.

[15] Stefanaki I., Foufa E., Tsatsou-Dritsa A., Dais P. (2003). Ochratoxin A concentrations in Greek domestic wines and dried vine fruits. Food Addit. Contam..

[16] Liao H., Lyon C.J., Ying B., Hu T. (2024). Climate change, its impact on emerging infectious diseases and new technologies to combat the challenge. Emerg. Microbes Infect..

[17] (2022). Commission Regulation (EU) 2022/1370 of 5 August 2022 Amending Regulation (EC) No 1881/2006 as Regards Maximum Levels of Ochratoxin A in Certain foodstuffs. Official Journal of the European Union (OJ L 206). https://eur-lex.europa.eu/eli/reg/2022/1370/oj/eng.

[18] Hing S., Narayan E.J., Andrew Thompson R.C., Godfrey S.S. (2016). The relationship between physiological stress and wildlife disease: Consequences for health and conservation. Wildl. Res..

[19] Armorini S., Altafini A., Zaghini A., Roncada P. (2016). Ochratoxin A in artisan salami produced in Veneto (Italy). Food Addit. Contam. Part B Surveill..

[20] Barrios-García M.N., Gonzalez-Polo M., Simberloff D., Classen A.T. (2023). Wild boar rooting impacts soil function differently in different plant community types. Biol. Invasions.

[21] Valente A.M., Acevedo P., Figueiredo A.M., Fonseca C., Torres R.T. (2020). Overabundant wild ungulate populations in Europe: Management with consideration of socio-ecological consequences. Mamm. Rev..

[22] Limonciel A., Jennings P. (2014). A review of the evidence that ochratoxin A is an Nrf2 inhibitor: Implications for nephrotoxicity and renal carcinogenicity. Toxins.

[23] Longobardi C., Ferrara G., Andretta E., Montagnaro S., Damiano S., Ciarcia R. (2022). Ochratoxin A and Kidney Oxidative Stress: The Role of Nutraceuticals in Veterinary Medicine—A Review. Toxins.

[24] Ramyaa P., Krishnaswamy R., Padma V.V. (2014). Quercetin modulates OTA-induced oxidative stress and redox signalling in HepG2 cells-up regulation of Nrf2 expression and down regulation of NF-κB and COX-2. Biochim. Biophys. Acta.

[25] Wang G., Zhang S., Lan H., Zheng X. (2024). Ochratoxin A (OTA) causes intestinal aging damage through the NLRP3 signaling pathway mediated by calcium overload and oxidative stress. Environ. Sci. Pollut. Res. Int..

[26] Beaulieu M., Costantini D. (2014). Biomarkers of oxidative status: Missing tools in conservation physiology. Conserv. Physiol..

[27] Costantini D., Helfenstein F. (2017). Editorial: Oxidative Stress and Signal Honesty. Front. Ecol. Evol..

[28] Sies H. (2015). Oxidative stress: A concept in redox biology and medicine. Redox Biol..

[29] Stoev S.D. (2022). New Evidences about the Carcinogenic Effects of Ochratoxin A and Possible Prevention by Target Feed Additives. Toxins.

[30] Khoi C.S., Chen J.H., Lin T.Y., Chiang C.K., Hung K.Y. (2021). Ochratoxin A-Induced Nephrotoxicity: Up-to-Date Evidence. Int. J. Mol. Sci..

[31] Więckowska M., Cichon N., Szelenberger R., Gorniak L., Bijak M. (2024). Ochratoxin A and Its Role in Cancer Development: A Comprehensive Review. Cancers.

[32] Damiano S., Longobardi C., De Marchi L., Piscopo N., Meucci V., Lenzi A., Ciarcia R. (2025). Detection of Ochratoxin A in Tissues of Wild Boars (Sus scrofa) from Southern Italy. Toxins.

[33] Kozlov A.V., Javadov S., Sommer N. (2024). Cellular ROS and Antioxidants: Physiological and Pathological Role. Antioxidants.

[34] Cordiano R., Di Gioacchino M., Mangifesta R., Panzera C., Gangemi S., Minciullo P.L. (2023). Malondialdehyde as a Potential Oxidative Stress Marker for Allergy-Oriented Diseases: An Update. Molecules.

[35] Son Y., Lee H.J., Ryu D., Kim J.R., Kim H.Y. (2024). Ochratoxin A induces hepatic and renal toxicity in mice through increased oxidative stress, mitochondrial damage, and multiple cell death mechanisms. Arch. Toxicol..

[36] Ighodaro O.M., Akinloye O.A. (2018). First line defence antioxidants-superoxide dismutase (SOD), catalase (CAT) and glutathione peroxidase (GPX): Their fundamental role in the entire antioxidant defence grid. Alex. J. Med..

[37] Schrenk D., Bignami M., Bodin L., Chipman J.K., Del Mazo J., Grasl-Kraupp B., Hogstrand C., Hoogenboom L.R., Leblanc J.C., EFSA Panel on Contaminants in the Food Chain (CONTAM) (2023). Risks for animal health related to the presence of ochratoxin A (OTA) in feed. EFSA J..

[38] Novi S., Vestuto V., Campiglia P., Tecce N., Bertamino A., Tecce M.F. (2023). Anti-Angiogenic Effects of Natural Compounds in Diet-Associated Hepatic Inflammation. Nutrients.

[39] Yang W.S., SriRamaratnam R., Welsch M.E., Shimada K., Skouta R., Viswanathan V.S., Cheah J.H., Clemons P.A., Shamji A.F., Clish C.B. (2014). Regulation of ferroptotic cancer cell death by GPX4. Cell.

[40] Wang G., Qin S., Zheng Y., Xia C., Zhang P., Zhang L., Yao J., Yi Y., Deng L. (2021). T-2 Toxin Induces Ferroptosis by Increasing Lipid Reactive Oxygen Species (ROS) and Downregulating Solute Carrier Family 7 Member 11 (SLC7A11). J. Agric. Food Chem..

[41] Li J., Cao F., Yin H.L., Huang Z.-J., Lin Z.-T., Mao N., Sun B., Wang G. (2020). Ferroptosis: Past, present and future. Cell Death Dis..

[42] Loboda A., Stachurska A., Sobczak M., Podkalicka P., Mucha O., Jozkowicz A., Dulak J. (2017). Nrf2 deficiency exacerbates ochratoxin A-induced toxicity in vitro and in vivo. Toxicology.

[43] Longobardi C., Damiano S., Fabroni S., Montagnaro S., Russo V., Vaccaro E., Giordano A., Florio S., Ciarcia R. (2024). Red Orange and Lemon Extract Ameliorates the Renal Oxidative Stress and Inflammation Induced by Ochratoxin A through the Modulation of Nrf2. Toxins.

[44] Simarro Doorten A.Y., Bull S., van der Doelen M.A., Fink-Gremmels J. (2004). Metabolism-mediated cytotoxicity of ochratoxin A. Toxicol. Vitr. Int. J. Public Assoc. BIBRA.

[45] Popescu R.G., Bulgaru C., Untea A., Vlassa M., Filip M., Hermenean A., Marin D., Țăranu I., Georgescu S.E., Dinischiotu A. (2021). The Effectiveness of Dietary Byproduct Antioxidants on Induced CYP Genes Expression and Histological Alteration in Piglets Liver and Kidney Fed with Aflatoxin B1 and Ochratoxin A. Toxins.

[46] Kuhn M., Hassan R., González D., Myllys M., Hobloss Z., Degen G.H., Humpf H.U., Hengstler J.G., Cramer B., Ghallab A. (2024). Role of albumin in the metabolism and excretion of ochratoxin A. Mycotoxin Res..

[47] Brown J.L., Rosa-Caldwell M.E., Lee D.E., Blackwell T.A., Brown L.A., Perry R.A., Haynie W.S., Hardee J.P., Carson J.A., Wiggs M.P. (2017). Mitochondrial degeneration precedes the development of muscle atrophy in progression of cancer cachexia in tumour-bearing mice. J. Cachexia Sarcopenia Muscle.

[48] Pfohl-Leszkowicz A., Manderville R.A. (2007). Ochratoxin A: An overview on toxicity and carcinogenicity in animals and humans. Mol. Nutr. Food Res..

[49] Gassó D., Vicente J., Mentaberre G., Soriguer R., Rodríguez R.J., Navarro-González N., Tvarijonaviciute A., Lavín S., Fernández-Llario P., Segalés J. (2016). Oxidative Stress in Wild Boars Naturally and Experimentally Infected with Mycobacterium bovis. PLoS ONE.

[50] Ferrara G., Longobardi C., D’Ambrosi F., Amoroso M.G., D’Alessio N., Damiano S., Ciarcia R., Iovane V., Iovane G., Pagnini U. (2021). Aujeszky’s Disease in South-Italian Wild Boars (Sus scrofa): A Serological Survey. Animals.

[51] Damiano S., Longobardi C., Ferrara G., Piscopo N., Riccio L., Russo V., Meucci V., De Marchi L., Esposito L., Florio S. (2023). Oxidative Status and Histological Evaluation of Wild Boars’ Tissues Positive for Zearalenone Contamination in the Campania Region, Southern Italy. Antioxidants.

[52] Dimatteo M., Di Napoli E., Paciello O., d’Aquino I., Iaccarino D., D’amore M., Guida M., Cozzolino L., Serpe F.P., Fusco G. (2024). Pathological Changes and CYP1A1 Expression as Biomarkers of Pollution in Sarpa Salpa and Diplodus Sargus. Animals.

[53] Damiano S., Andretta E., Longobardi C., Prisco F., Paciello O., Squillacioti C., Mirabella N., Florio S., Ciarcia R. (2020). Effects of curcumin on the renal toxicity induced by ochratoxin A in rats. Antioxidants.

[54] Damiano S., Longobardi C., Andretta E., Prisco F., Piegari G., Squillacioti C., Montagnaro S., Pagnini F., Badino P., Florio S. (2021). Antioxidative Effects of Curcumin on the Hepatotoxicity Induced by Ochratoxin A in Rats. Antioxidants.

[55] Melini S., Pirozzi C., Lama A., Comella F., Opallo N., Del Piano F., Di Napoli E., Mollica M.P., Paciello O., Ferrante M.C. (2024). Co-Micronized Palmitoylethanolamide and Rutin Associated with Hydroxytyrosol Recover Diabesity-Induced Hepatic Dysfunction in Mice: In Vitro Insights into the Synergistic Effect. Phytother. Res..

